# Immunohistochemical and ultrastructural features of congenital melanocytic naevus cells support a stem-cell phenotype

**DOI:** 10.1111/bjd.12323

**Published:** 2013-08-13

**Authors:** VA Kinsler, G Anderson, B Latimer, D Natarajan, E Healy, GE Moore, NJ Sebire

**Affiliations:** 1 Paediatric Dermatology, Great Ormond Street Hospital for ChildrenLondon, WC1N 3JH, U.K; 2 Clinical and Molecular Genetics Unit, UCL Institute of Child HealthLondon, U.K; 3 Paediatric Histopathology, Great Ormond Street Hospital for ChildrenLondon, WC1N 3JH, U.K; 4 Neural Development Unit, UCL Institute of Child HealthLondon, U.K; 5 Dermatopharmacology, University of SouthamptonSouthampton, U.K; 6 Cancer Biology Unit, UCL Institute of Child HealthLondon, U.K

## Abstract

**Background** Multiple congenital melanocytic naevi (CMN) in one individual are caused by somatic mosaicism for *NRAS* mutations; however, the lineage of the mutated cells remains uncertain.

**Objectives** To test the hypothesis that CMN may be derived from cutaneous stem cells.

**Methods** Sixty-six CMN samples from 44 patients were stained for immunohistochemical (IHC) markers of melanocytic differentiation (TYR, TRP1, TRP2, LEF1, MITF, cKit), pluripotency (nestin, fascin, CD133, CD20, CD34), monocyte/macrophage lineage (CD68, CD163, CD14), proliferation (Ki67) and MTOR/Wnt-signalling pathway activation (pS6, β-catenin). Semiquantitative scoring compared samples with naevus cell nesting (group 1) with those with only diffuse dermal infiltration (group 2). Transmission electron microscopy (TEM) was performed on 10 samples.

**Results** A normal melanocyte population was seen overlying many dermal CMN. Group 1 samples were significantly more likely to express melanocytic differentiation markers than group 2, and expression decreased significantly with depth. Expression of these markers was correlated with each other, and with nestin and fascin. CD20 staining was positive in a substantial proportion and was stronger superficially. Expression of β-catenin and pS6 was almost universal. Some samples expressed monocyte/macrophage markers. TEM revealed variable naevus cell morphology, striking macromelanosomes, double cilia and microvilli.

**Conclusions** Congenital melanocytic naevi development frequently coexists with normal overlying melanocyte development, leading us to hypothesize that in these cases CMN are likely to develop from a cell present in the skin independent of, or remaining after, normal melanocytic migration. IHC and TEM findings are compatible with CMN cells being of cutaneous stem-cell origin, capable of some degree of melanocytic differentiation superficially.

## What’s already known about this topic?

- The cell of origin of congenital melanocytic naevi (CMN) is not known.
- Theoretical candidates proposed include mature basal layer melanocytes, direct precursors of the melanocytes destined for the basal layer (melanoblasts), or stem cells residing within the dermis.
- In recent years stem cells have been isolated from human hair follicles, and from non-hair-bearing dermis.

## What does this study add?

- A normal melanocyte population overlies many dermal CMN, leading us to hypothesize that in these cases CMN are likely to develop from a cell present in the skin independent of, or remaining after, normal melanocytic migration.
- Immunohistochemistry and transmission electron microscopy of CMN cells have identified stem-cell characteristics, with differentiation towards melanocytes in the superficial dermis.
- These findings support the hypothesis that the cell of origin of CMN could be a cutaneous stem cell.

Individuals with multiple congenital melanocytic naevi (CMN) and/or neurocutaneous melanosis have recently been shown to be mosaic for mutations at codon 61 of *NRAS*.^1^ However, the lineage of the cell sustaining the initial postzygotic mutation is not yet known. While studies of cellular morphology and immunophenotype cannot determine cell lineage alone, they can support or refute evidence for hypotheses regarding lineage.

It is not yet clear whether congenital and acquired melanocytic naevus (AMN) cells develop from the same cell type; however, fundamental similarities between naevus cells suggest that they may. Indeed, the most reliable histological features for distinguishing CMN from AMN are related to the distribution of naevus cells, and size and depth of the naevus, rather than factors pertaining to individual cell morphology.^2^–^3^ Another clue to lineage is the striking variation in cellular morphology and immunostaining both with depth of lesional cells in CMN and AMN, and within different areas of larger CMN. For example, while the production of melanin by superficial naevus cells is suggestive of a melanocytic lineage, this is generally not a feature of deeper naevus cells. Similarly, nest formation and lack of dendrites argue against typical melanocyte behaviour. More dramatic is the so-called ‘neurotization’ seen in a significant number of large CMN, in which naevus cells in the deeper areas morphologically resemble Schwann cells or even form Schwannomas.^4^–^5^ More rarely CMN can contain areas of chondroid foci^6^ or neurocristic hamartoma.^7^

Many theories of naevogenesis have been proposed (for a review see Krengel).^8^ Recent hypotheses have suggested a possible stem-cell origin for naevus cells, thereby producing a unifying theory of congenital and acquired naevogenesis (at least with respect to cell lineage), as cutaneous stem cells could be present from fetal development through to adult life. One such was proposed by Cramer, who suggested neural-sheath stem cells as a candidate, based on the anatomical and temporal relationships between naevus cells and developing nerves, and histopathological examination of fetal skin for occult melanocytic lesions.^9^,^10^ In some support of this theory is the recent discovery that there is a second anterior route of melanocyte precursor migration from the neural crest in mice,^12^ in addition to the classical dorsolateral route. *In vitro* studies of Schwann cells demonstrate their potential to generate melanocytes under the right conditions.^13^,^14^ However, as yet no nerve sheath stem cells have been isolated from human dermis. Furthermore, from a clinical perspective, if the transformation from neural-sheath stem cell to naevus cell could occur at any point along the development of the nerve as suggested, we would expect to see CMNs at least occasionally in a single complete dermatome and this has not been described.

An alternative theory of CMN derivation from stem cells has been proposed by Barnhill *et al*.,^16^ who suggested a neural-crest stem cell migrating to the skin along blood vessels as the cell of origin, based on histopathological evidence of angiotropism of naevus cells, and parallels with angiotropism in melanoma.^16^–^17^ However, again no direct evidence of such a cell population exists as yet in human dermis and this has not been described.

Finally, it was suggested by Krengel in 2005 that melanocytic naevi could arise from hair-follicle stem cells, which had then been identified in mice.^8^ In contrast to the hypotheses presented above there is now a well-characterized population of neural-crest stem cells in both mice and humans residing in the bulge area of the hair follicle.^18^–^21^ In mice, a subset of these has been designated melanocyte stem cells (MSCs), which have been differentiated from other stem cells within the bulge by their antigenic signature and their ability to generate mature melanocytes. The specific markers of MSC in the hair-follicle bulge in mice are TRP2+, PAX3+, TYR−, TRP1−, MITF−, cKit−, LEF1−, SOX10− and Ki67−. All these markers become positive once the cells are in the transamplifying state, and remain unchanged in fully differentiated melanocytes.^22^ Hair-follicle bulge neural-crest stem cells from humans have also been found to generate melanocytes (along with many other cell types),^23^ although a specific MSC subpopulation has not so far been defined.

Another population of dermal stem cells has been isolated from both mice and humans,^24^ termed skin-derived precursors (SKP), which are distinct from MSCs in mice by the formation of floating spheres in culture rather than an adherent monolayer, and by the absence of expression of TRP2.^25^,^26^ SKP can be induced to differentiate into both neural and mesodermal progeny, including neurons, glia, smooth-muscle cells and adipocytes, but melanocytic differentiation has not been attempted. One niche for these cells is thought to be the dermal papilla of the hair follicle;^27^ however, they have also been isolated from non-hair-bearing skin,^25^ indicating at least one other unidentified niche within the dermis.^24^ They have been shown in mice to be present from embryogenesis through to adulthood.^27^

This study aimed to test the hypothesis that CMN cells may be derived from one of the currently identified and defined populations of cutaneous stem cells, using systematic characterization of the antigen expression profile and electron microscopic appearances of a series of CMN.

## Materials and methods

### Subjects and samples

This study was approved by the Research Ethics Committee and Research and Development office of Great Ormond Street Hospital and the University College London Institute of Child Health. Samples examined were surplus to diagnostic needs where tissue had been obtained for clinical indications, as part of routine patient care. Forty-four patients were selected from the histopathology database of children who had CMN tissue removed, on the basis of adequate clinical data, including projected adult size (PAS) of the main lesion (Table S1; see Supporting Information) and the total number of naevi. In total, 42/44 patients had multiple CMN, defined as two or more lesions present at birth. Samples were also specifically selected for the absence of proliferative nodules or melanoma. Sixty-six formalin-fixed paraffin-embedded CMN samples from 44 patients were included. Single samples were available for 23 patients, two samples for 20, and three samples for one patient. Each sample was classified by whether there was a prominent superficial nesting pattern on routine haematoxylin and eosin examination (group 1, 31 samples), or only a diffuse dermal infiltration without nesting (group 2, 35 samples). More than one sample from a single patient was included where the morphological pattern varied in different areas of the same block, to allow for comparison within an individual. Tissue arrays were then made from these areas of the blocks to allow uniform immunostaining of large numbers of samples simultaneously. A sample each of normal skin, AMN and malignant melanoma were included on each array as controls.

Clinical phenotyping had been performed using an estimation of PAS of the largest CMN, and an estimate of the total number of naevi, which is the most widely used method of classification.^28^

### Immunohistochemistry

Sections 4 μm in size were cut, and immunostaining was performed using standard protocols on the automated Leica BOND-MAX immunostainer (Leica Biosystems, Newcastle Upon Tyne, U.K.). All antibodies were optimized for use on paraffin sections with appropriate positive and negative controls (Table 1). Antibodies used were markers of melanocyte differentiation [TYR, TRP1, TRP2, MITF, cKit, LEF1], of pluripotency (nestin, fascin, CD133, CD20, CD34), of monocyte/macrophage lineage (CD68, CD163, CD14), of proliferation (Ki67) and of mammalian target of rapamycin (mTOR) and Wnt-signalling pathway activation (pS6 and β-catenin, respectively). Monocyte/macrophage lineage markers were included because of fascin positivity in many sections (a monocyte/macrophage marker and stem-cell marker). Immunohistochemical results were scored blindly and semiquantitatively, using a scale of 0–3 for intensity, with patterns of staining in superficial vs. deep dermal cells also noted. Where staining superficially and more deeply within the dermis was clearly different these areas were scored separately. Stains were assessed in conjunction with haematoxylin and eosin sections to take into account background melanin pigmentation in all cases.

**Table 1:** Details of antibodies used for immunohistochemical staining

Antibody	Target protein description	Cellular location	Most commonly used as a marker for	Dilution	Manufacturer	Antigen retrieval
c-Kit	Receptor tyrosine kinase and factor	Cell membrane/ cytoplasmic	Mast cells, melanocytes	1 : 40	Leica	HIER20 pH9
LEF1	Transcriptio*n* factor	Cytoplasmic/nuclear	Melanocytes, Wnt pathway, hair follicle	1 : 100	Abcam	HIER20 pH6
MITF	Transcriptio*n* factor	Cytoplasmic/nuclear	Melanocytes	1 : 1000	Abcam	HIER20 pH6
TRP1	Melanosomal enzyme	Cytoplasmic	Melanocytes	1 : 200	Abcam	HIER20 pH9
TRP2	Melanosomal enzyme	Cytoplasmic	Melanocytes	1 : 1000	Abcam	HIER20 pH9
Tyrosinase	Melanosomal enzyme	Cytoplasmic	Melanocytes	1 : 200	Abcam	HIER20 pH9
Nestin	Intermediate filament protein	Cytoplasmic	Neural stem cells, other stem cells	1 in 2000	Chemikon	HIER20:ER1
Fascin	Actin bundling protein	Cytoplasmic	Macrophage/monocyte lineage, stem cells	1 in 200	Novocastra	HIER20:ER1
CD68	Glycoprotein which binds to low density lipoprotein	Cell membrane/ cytoplasmic	Macrophage/monocyte lineage	1 in 200	Dako	Ag Retrieval Enzyme 1 : 10 min
CD163	Receptor; clearance of Hb/haptoglobin complexes in macrophages	Cell membrane/ cytoplasmic	Macrophage/monocyte lineage	1 in 50	Dako	HIER20:ER1
CD14	Receptor binding bacterial lipopolysaccharide, part of innate immunity	Cell membrane/ cytoplasmic	Macrophage/monocyte lineage	1 in 25	Dako	HIER20:ER1
CD133	Glycoprotein in cellular protrusions	Cell membrane/ cytoplasmic	Neural, glial and adult stem cells	1 in 25	MACS	HIER20:ER2
CD20	Calcium channel, optimises B cell function	Cell membrane/ cytoplasmic	B cell lineage, melanoma stem cells	1 in 1000	DAKO	HIER20:ER2
CD34	Sialomucin protein	Cell membrane/ cytoplasmic	Haematopoietic, vascular cells + mesenchymal stem cells		Leica ready made	HIER20:ER2
Ki67	Necessary for cell proliferation	Nuclear	Proliferation index		Leika ready made	HIER20:ER2
Β-catenin	Component of adherens junctions, anchors actin cytoskeleton	Nuclear; cytoplasmic in specific situtations	Wnt pathway activation	1 in 100	Dako	HIER20:ER1
pS6	Ribosomal protein	Cytoplasmic	MTOR pathway activation	1 in 50	Cell Signalling	HIER30:ER2

HIER20, heat induced epitope retrieval for 20 min; ER1, epitope retrieval solution 1; ER2, epitope retrieval solution 2.

While it is not possible to be certain that the markers used in the current study are unequivocally indicating the pathways and processes they are most commonly associated with, we have attempted to use markers used in previous publications such as those referenced herein.

The number of samples staining positively in groups 1 and 2 were compared using Fisher’s exact test. The mean intensity of staining between the groups was compared using the independent *t*-test. Correlations between patterns of staining were with two-tailed Pearson correlation.

### Electron microscopy

Transmission electron microscopic examination was performed on tissue obtained specifically for research, from patients having routine surgery under general anaesthetic for removal of part of a CMN. Written consent was obtained in all cases. Three samples were taken from each of four patients: from the largest CMN, from a smaller separate CMN (all of which were < 5 cm PAS) and from macroscopically uninvolved skin. Samples of the main CMN only were obtained from two additional patients. All six patients were severely affected as defined by the size and number of lesions (Table S2; see Supporting Information). For each biopsy a small piece of fresh tissue was fixed in 2·5% glutaraldehyde in 0·1 mol L^−1^ cacodylate buffer with secondary fixation in 1·0% osmium tetroxide. Tissues were dehydrated in graded ethanol, transferred to propylene oxide and finally infiltrated and embedded in Agar 100 epoxy resin (Agar Scientific, Stansted, U.K.). Next, 90-nm ultrathin sections were cut using a DiATOME diamond knife (DiATOME, Hatfield, PA, U.S.A.) on a Leica Ultracut UCT Ultramicrotome (Leica Microsystems, Milton Keynes, U.K.). Sections were picked up on copper grids and stained with alcoholic uranyl acetate and Reynolds lead citrate. Ultrastructural examination was performed using the JEM-1400 120 kV Transmission Microscope [JEOL (U.K.) Ltd, Welwyn Garden City, U.K.].

## Results

### Immunohistochemistry

**Figure 1:**
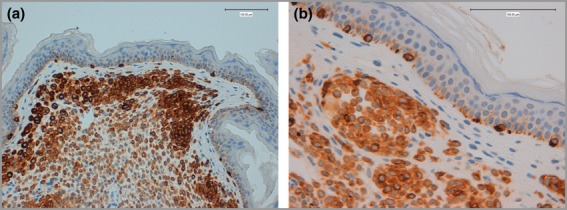
Staining for tyrosinase in two different samples; (a) ×20, scale bar 100 μm; (b) ×40, scale bar 50 μm; showing a normal melanocyte population along the basement membrane overlying dermal congenital melanocytic naevi.

A normal melanocyte population was visible in the basal layer overlying many dermal CMN. This was particularly highlighted on staining for TYR (Fig. 1), which is expressed by both normal melanocytes and upper dermal naevus cells. In samples where there was junctional involvement it was not possible to assess whether there was a normal melanocyte population due to the proliferation of naevus cells within the basal layer. Frequently there was a mixed pattern within a sample, where some areas involved the junction and some clearly showed a normal melanocyte population.

**Figure 2:**
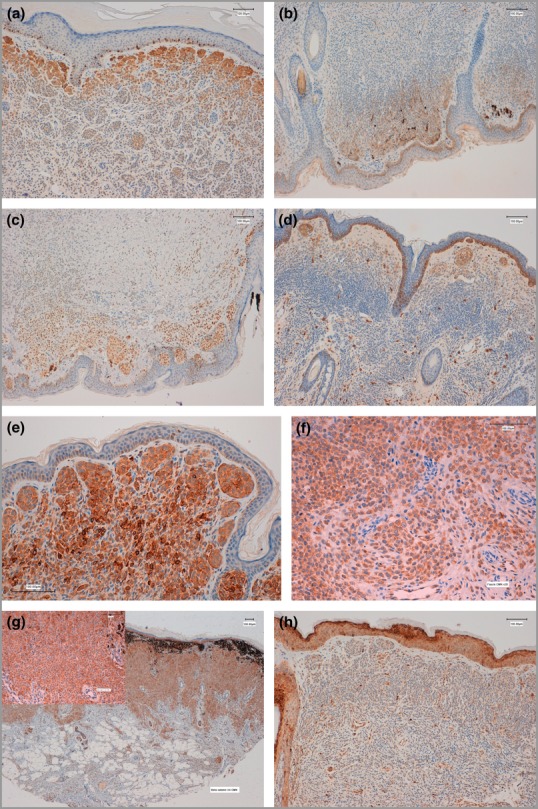
Positive staining for melanocytic differentiation markers, (a) TRP1 (b) MITF (c) LEF1 and (d) cKit, all showing increased staining superficially and little or no staining deeply. Staining for these markers was generally reduced in samples without a nesting pattern. Staining for (e) nestin, (f) fascin, (g) β-catenin (cytoplasmic) and (h) pS6 showed less variability with depth of the lesional cells.

There were two consistent patterns of staining regarding the melanocyte differentiation markers TRP1, TRP2, LEF1, MITF and cKit. Firstly, these markers were more likely to be positive in the samples displaying nest formation (group 1) than in those with diffuse infiltration only (group 2; Table 2, Fig. 2). Secondly, in positive samples in both groups there was a significant decrease in staining intensity with increasing dermal depth (Table 2; Fig. 2). This pattern suggests an increase in differentiation towards a mature melanocyte phenotype in those samples that show nesting behaviour, and in more superficial cells within most CMN. TYR staining had similar but nonsignificant differences between the groups.

**Table 2:** Immunohistochemical staining results in 66 congenital melanocytic naevi samples. For antibodies showing a marked difference in intensity of staining between superficial and deep levels of the naevus, intensity scores are given for both. Where the sample number is less than 31 in group 1 or < 35 in group 2 this was due to individual tissue samples not being sufficient for particular stains. Where staining was different in superficial and deep levels of the samples scoring of these areas was done separately

Antigen	Group 1^a^	Group 2^a^	*P*-value^b^	Group 1 intensity scores, superficial/deep; *P*-value^c^	Group 2 intensity scores, superficial/deep; *P*-value^c^	Unpaired *t*-test *P*-values, superficial/deep^c^
TYR	29/30	30/33	0·614	2·33 (0·130)/0·97 (0·140); < 0·001**	2·00 (0·174) /0·76 (0·169); < 0·001**	0·136/0·349
TRP1	31/31	28/34	0·025*	2·48 (0·102)/1·13 (0·166); < 0·001**	1·74 (0·181)/0·50 (0·114); < 0·001**	0·001**/0·002**
TRP2	29/30	27/34	0·058	1·97 (0·122)/0·80 (0·139); < 0·001**	1·26 (0·154)/0·18 (0·079); < 0·001**	0·001**/< 0·001**
MITF	12/18	15/15	0·021*	2·07 (0·118)/1·47 (2·15); 0·007**	1·06 (0·206)/0·78 (0·207); 0·056	< 0·001**/0·029*
LEF1	26/29	20/35	0·005**	1·83 (0·165)/0·66 (0·188); < 0·001**	0·94 (0·158)/0·2 (0·069); < 0·001**	< 0·001**/0·017*
cKit	30/30	20/34	< 0·001**	2·30 (0·145)/1·17 (0·160); < 0·001**	0·91 (0·148)/0·38 (0·104); < 0·001**	< 0·001**/< 0·001**
Nestin	19/31	20/34	1·000	0·65 (0·099)	0·68 (0·109)	0·834
Fascin	23/31	23/35	0·593	1·55 (0·201)	1·34 (0·188)	0·458
CD68	0/31	2/35	0·492	N/A	N/A	N/A
CD163	0/31	2/35	0·492	N/A	1·00 (0)	N/A
CD14	0/31	2/35	0·492	N/A	2·00 (0)	N/A
CD133	0/0	0/0	1·000	N/A	N/A	N/A
CD20	10/30	18/33	0·129	0·4 (0·113)/0·1 (0·074); 0·005**	0·76 (0·138)/0·36 (0·114); 0·002**	0·052/0·061
CD34	0/0	0/0	1·000	N/A	N/A	N/A
Ki67	< 1% positivity	< 1% positivity except three samples > 10%	N/A	N/A	N/A	N/A
β-Catenin	31/31	33/33	1·000	2·58 (0·101)/0·71 (0·175); < 0·001**	1·82 (0·127)/0·21(0·084); < 0·001**	< 0·001**/0·011*
pS6	30/30	34/35	1·000	1·83 (0·118)	1·22 (0·129)	0·002*

TYR, tyrosinase; TRP, tyrosinase-like protein; MITF, microphthalmia-associated transcription factor; LEF, lymphoid enhancer-binding factor; N/A, not applicable.

* Significant at 0·05 level.

** Significant at 0·01 level.

a Number of positive samples/total number of samples stained.

b Fisher’s exact *P*-values for comparison of number of positive samples between groups 1 and 2.

c Mean (SEM) intensity score for superficial staining/deep staining; *P*-value for paired *t*-test comparison of means of superficial and deep.

d Comparison of intensity of staining between groups 1 and 2, superficial/deep (where applicable).

**Figure 3:**
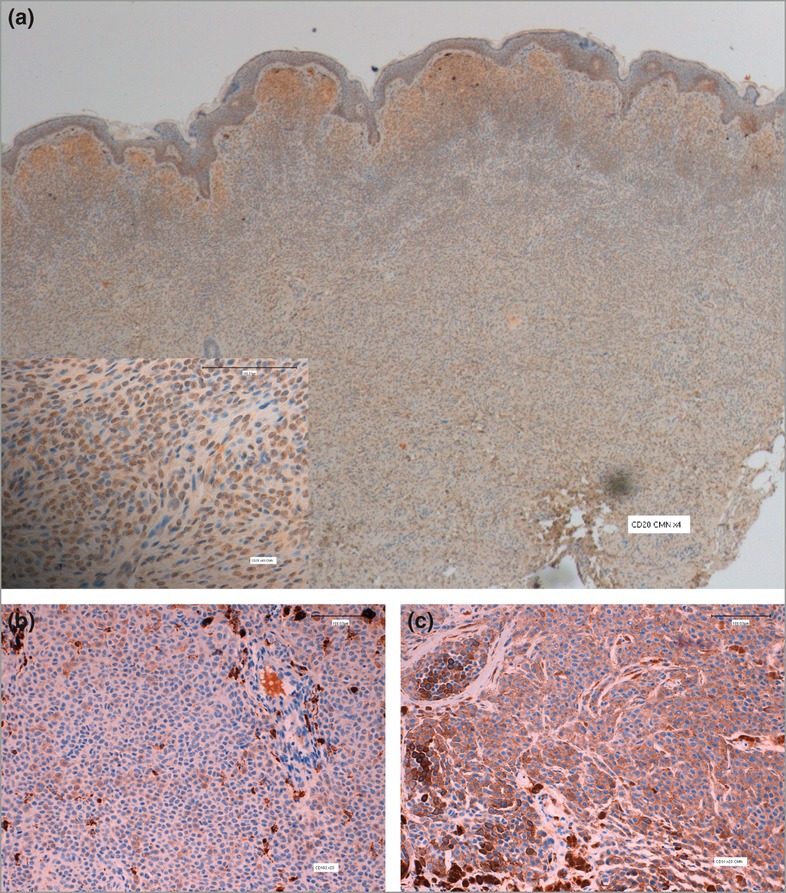
Congenital melanocytic naevi samples without a nesting pattern showed (a) CD20 staining in the majority (main picture ×4, scale bar 100 μm; inset from area within black square ×40, scale bar 50 μm); in a small subset (b) CD163 staining naevus cells and dermal dendritic cells (×20, scale bar 100 μm) and (c) CD14 staining naevus cells (×20, scale bar 100 μm).

The stem-cell markers nestin and fascin were positive in the majority of samples, but these showed little variation with the depth of the lesion, and no difference between the groups (Table 2, Fig. 2). The B-cell/stem-cell marker CD20 was positive in a substantial but similar proportion of samples in both groups, and staining was stronger superficially (Fig. 3). CD133 and CD34 were negative in all samples.

Expression of pS6, a marker of mTOR pathway activation, was almost universal and showed little variation with depth, but was significantly stronger in intensity in group 1 than group 2. Cytoplasmic β-catenin was again almost universally expressed more strongly in group 1, and in both groups was stronger superficially (Table 2, Fig. 2). The Ki67 proliferation index was generally low, approximately 1% in the majority of samples, in a pattern of evenly spaced cells within the deep dermis. Two samples also showed expression of the macrophage/monocyte lineage markers CD163 and CD14, but were negative for CD68, and also showed an increased proliferation index of around 10% (Fig. 3). These two samples were not morphologically distinguishable from the others on haematoxylin and eosin examination, and although they were both from individuals with the largest CMN (> 60 cm PAS) it is not yet clear what separates this small group with a specific immunophenotype. Two other samples from group 2 expressed CD68 on naevus cells, but were negative for CD14 and CD133.

Comparison of samples from the same individual but from different groups did not show consistency of staining between samples (Table S3; see Supporting Information). Correlation of staining patterns revealed highly significant correlations between melanocytic differentiation markers, and with nestin and fascin expression (all significant at the *P *<* *0·001 level), but no correlation with CD20 expression.

### Electron microscopy

**Figure 4:**
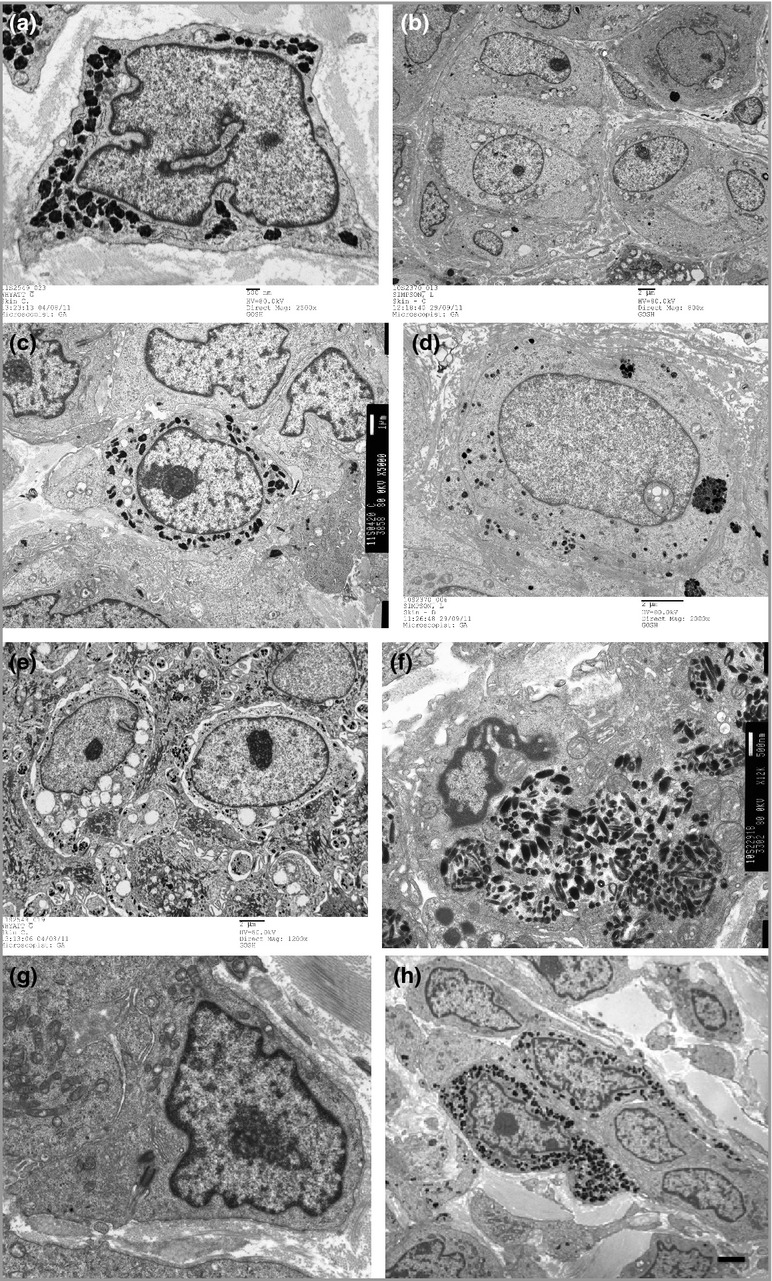
Transmission electron micrographs of congenital melanocytic naevus cells; (a) naevus cell showing large nucleus with indentations, melanosomes in cytoplasm producing melanin, ×2500, scale bar 500 nm; (b) naevus cells in a nest encased in basal lamina (arrow), ×800, scale bar 2 μm; (c) variability in melanin production between neighbouring cells, ×5000, scale bar 1 μm; (d) inclusion body in nucleus (arrow), ×2000, scale bar 2 μm; (e) inclusion bodies in cytoplasm, and microvilli (arrow), ×1200, scale bar 2 μm; (f) macromelanosome complex, ×12 000, scale bar 500 nm; (g) double cilia seen as parallel lines (arrow), scale bar 500 nm; (h) cluster of naevus cells in macroscopically normal skin, ×3000, scale bar 2 μm.

Examination of CMN tissue showed normal epidermis in all cases. Naevus cells had large nuclei, often with irregular edges and indentations (Fig. 4a). In the dermis, naevus cells were abundant and in close proximity to each other, even when not in nests. Nesting cells appeared to have primitive junctions between them, and the nests were clearly encased in a basal lamina (Fig. 4b). Naevus cells were seen clustered around adnexae but none were seen in the walls or lumina of blood vessels or lymphatics, and there was no evidence of unusual relationships with nerves. Melanization was very variable between cells in the same individual, and even in the same electron microscope field (Fig. 4c), mirrored by variable presence of melanosomes, which were often in large collections, constituting macromelanosomes (Fig. 4f). Centrioles and inclusion bodies were seen in the nucleus (Fig. 4d), but no multiple centrioles. True dendrites were rarely observed, but many naevus cells exhibited microvilli (Fig. 4e). Vacuoles in the cytoplasm were common. Cilia were visualized and were occasionally double (Fig. 4g). The ultrastructural findings in the smaller naevi from the same patients were indistinguishable from those of their largest lesions, showing more variation between patients than between different naevi in the same patient. Macroscopically normal skin biopsy specimens were ultrastructurally normal in three of four cases. However, in one case there was clear evidence of naevus cells in the dermis of the normal skin, some of which were producing melanin (Fig. 4h). Naevus cells were not identifiable on haematoxylin and eosin sections from the same sample despite re-review.

## Discussion

This study provides additional data examining the hypothesis that CMN cells are derived from cutaneous stem cells, using a panel of melanocytic differentiation and stem-cell markers, and electron microscopy. This is highly topical as there is increasing interest in the possible role of cutaneous stem cells in many skin tumours, including melanoma.^29^–^32^ Melanomas have been found to express the stem-cell markers nestin, CD166, CD133, ATP-binding cassette subfamily B member 5 and CD20,^29^,^33^ leading to characterization of subpopulations of ‘melanoma stem cells’, and the proposal that melanoma could arise from dermal stem cells rather than mature melanocytes.^31^–^35^ Furthermore, altered expression of certain stem-cell markers in melanoma, such as increased nestin,^36^–^37^ decreased fascin^38^–^39^ and CD133 positivity,^30^ have been associated with increased malignant potential and metastasis.

The first important observation in the present study was that of an apparently normal epidermal melanocyte population in the basal layer overlying many dermal CMNs (Fig. 1). This could be interpreted in various ways: (i) either a mutation takes place in a cell that is present in the skin independent of, or remaining after, normal melanocytic migration, but which is capable of differentiation towards the melanocytic lineage; (ii) the naevus develops from a single mature epidermal melanocyte after basal layer melanocyte development has finished, leaving the other mature melanocytes in place; or (iii) the naevus develops from one cell of a committed melanoblast population but the resultant defect is then covered by the normal development of surrounding melanoblasts. Both of the latter two propositions seem unlikely in the context of vast bathing-trunk CMNs covering 80% of the body surface area, supporting the first proposal rather more in the authors’ minds; however, this is only a hypothesis at this stage. It is not possible to comment on the development of mature melanocytes in CMNs with a junctional component, and we can therefore not discount that these CMNs could have a different cell of origin.

The second observation is that superficial CMN cells are more differentiated towards normal melanocytes than deeper dermal cells, echoing the findings of previous authors using different antigenic markers.^40^–^41^ Samples with nesting are also more strongly differentiated towards melanocytes than those without. Despite this, the retention of stem-cell markers even in the superficial nesting cells could be supportive of a stem-cell origin hypothesis. This could also be compatible with the lack of correlation between antigen expression in different parts of a sample from the same patient, in that the cells within different microenvironments appear to be able to adapt their phenotype.

mTOR activity in CMNs has not been studied previously, but consistent pS6 expression would be consistent with causal *NRAS*-activating mutations in the majority of multiple CMNs,^1^ as *NRAS* is an upstream component of the mTOR pathway. Expression of pS6 has also been documented in the majority of cutaneous melanomas, although interestingly AMNs in that study were only rarely positive.^42^ The sample of AMNs included in our arrays showed expression of pS6.

Two samples expressed the monocyte/macrophage lineage markers CD163 and CD14, and two others CD68. This finding suggests that it is possible for some CMNs to show evidence of either further dedifferentiation, or differentiation towards other lineages. These markers have been found in one study of melanoma, where 35% of samples were positive for CD163, and 10% positive for CD68.^43^

The largest previous studies of the ultrastructural features of CMN reported irregular and indented nuclei, complex dendrites, nuclear inclusions, scattered large clusters of melanosomes, increased numbers of cilia and centrioles, contact between naevus cells and nerve cells, and naevus cells in both the walls and lumina of blood vessels and lymphatics.^44^,^45^ We have confirmed the findings of irregular indented nuclei of double cilia, although this was not a universal feature, and of nuclear inclusions and large abnormal collections of melanosomes. Furthermore we have shown that nests can be surrounded by a basal lamina, which may suggest the development of the nest from a single dividing cell, and that even non-nested cells appear to have primitive junctions between them. All these features would be compatible with a stem-cell phenotype with partial melanocytic differentiation.

In one patient, electron microscopic examination revealed typical naevus cells in the dermis of a macroscopically normal skin sample. This has been described in patients without CMN where microscopic aggregates of naevus cells were found in 1% of macroscopically normal skin samples after excisions for nonmelanocytic skin tumours.^47^ The presence of naevus cells in normal skin may therefore represent a newly formed naevus about to emerge, or it may imply that CMN cells are widespread within the skin from birth, and only large enough aggregates are visible.

**Figure 5:**
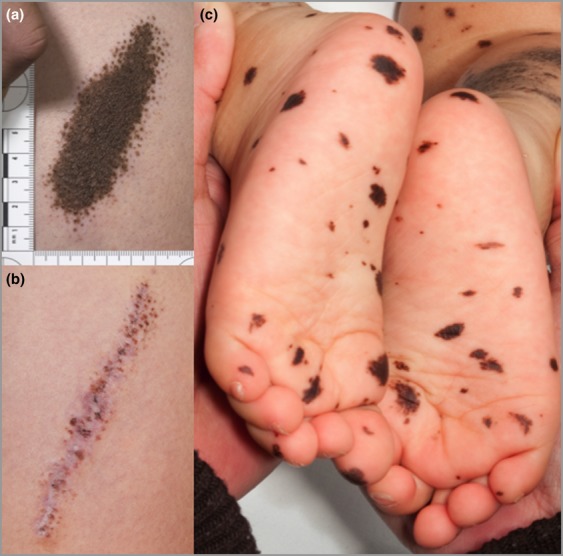
(a) Perifollicular patterning of pigmentation at the borders of a single congenital melanocytic naevus (CMN) pre-resection, and (b) perifollicular regrowth after total macroscopic resection, suggestive of a hair follicle-associated reservoir of naevus cells; (c) palmoplantar CMNs are common in individuals with multiple CMNs, indicating that CMNs can also arise in areas devoid of hair follicles.

In conclusion, the findings in this study are supportive of the hypothesis that CMN cells could develop from stem cells in the skin, which have at least partial melanocytic differentiation potential. Considering which of the currently identified populations of stem cells this could be, we feel there is clinical evidence that at least some CMNs may derive from cells closely related to the hair follicle. Figure 5a shows regeneration of CMN after resection, a phenomenon that occurs in a substantial proportion of cases.^48^ In this case, both the edges of the original lesion and the repigmentation within the scar show perifollicular regeneration of naevus, reminiscent of the well-known pattern of repigmentation in vitiligo (Fig. 5a,b). However, this follicular pattern is not seen in most CMNs, and there are good clinical signs supportive of a nonfollicular cell of origin, in particular the frequent development of CMN on nonhair-bearing palmoplantar surfaces in patients with multiple CMNs (Fig. 5c). Immunohistochemically, our findings suggest that CMN cells are more aligned with the characterization of SKP cells than the murine MSCs, as they frequently fail to express TRP2 in deeper layers, and can express antigens as varied as CD20, CD68, CD14 and CD133.

## Supporting Information

**Table S1.** Clinical phenotype of the patients who provided samples in groups 1 and 2. **Table S2.** Detailed phenotype of children for electron microscopic studies. **Table S3.** Examples of the intraindividual variability of intensity scores in different samples from the same patient.
