# Supplementary material for: Immunohistochemical and ultrastructural features of congenital melanocytic naevus cells support a stem-cell phenotype
Source: Br J Dermatol. 2013 Aug 13;169(2):374–83. doi: 10.1111/bjd.12323 (PMC3838625; doi:10.1111/bjd.12323)
Supplement: Supplementary file 1 — Table S1. Clinical phenotype of the patients who provided samples in groups 1 and 2. Table S2. Detailed phenotype of children for electron microscopic studies. Table S3. Examples of the intraindividual variability of intensity scores in different samples from the same patient. [file bjd0169-0374-sd1.docx]

**Supplementary Table 1**

Clinical phenotype of the patients who provided samples in groups 1 and 2. Samples in group 1 showed nesting on H+E with or without areas of diffuse infiltration, whereas samples in group 2 had only diffuse dermal infiltration.

| **PAS of largest CMN** | **Group 1 n = 31 (%)** | **Group 2 n = 35 (%)** |
| --- | --- | --- |
| <10cm | 22 | 11 |
| 10-20cm | 17 | 10 |
| 20-40cm | 13 | 14 |
| 40-60cm | 13 | 29 |
| >60cm | 35 | 36 |

**Supplementary Table 2**

Detailed phenotype of children for Electron Microscopic studies

| **Patient number** | **PAS main CMN** | **Total number of naevi** | **Site of main naevus** | **Age at biopsy (yrs)** |
| --- | --- | --- | --- | --- |
| 1 | 40-60cm | 50-100 | Back, buttocks | 14 |
| 2 | >60cm | >200 | Back, buttocks, thighs, abdo | 7 |
| 3 | 20-40cm | 100-200 | Scalp | 10 |
| 4 | >60cm | 100-200 | Back, buttocks, thighs, abdo | 9 |
| 5 | No single larger lesion | 100-200 | Not applicable | 1 |
| 6 | >60cm | >200 | Face, scalp, neck, upper back | 15 |

**Supplementary Table 3**

Examples of the intra-individual variability of intensity scores in different samples from the same patient. Intensity scores are graded 0-3. Three patients (six samples) and five antibodies are shown as an example.

| **Case number** | **Sample number** | **TRP1 superficial** | **TRP1 deep** | **TRP2 superficial** | **TRP2 deep** | **LEF1 superficial** | **LEF1 deep** | **cKit superficial** | **cKit deep** |
| --- | --- | --- | --- | --- | --- | --- | --- | --- | --- |
| 1 | 1 | 3 | 0 | 3 | 0 | 2 | 0 | 1 | 1 |
| 1 | 2 | 3 | 3 | 3 | 3 | 3 | 3 | 3 | 3 |
| 2 | 1 | 2 | 1 | 1 | 0 | 1 | 1 | 2 | 2 |
| 2 | 2 | 0 | 0 | 0 | 0 | 0 | 0 | 0 | 0 |
| 3 | 1 | 3 | 1 | 2 | 0 | 2 | 0 | 3 | 1 |
| 3 | 2 | 2 | 0 | 2 | 0 | 2 | 0 | 2 | 1 |
